# Correction: Alectinib (CH5424802) antagonizes ABCB1- and ABCG2-mediated multidrug resistance in vitro, in vivo and ex vivo

**DOI:** 10.1038/s12276-020-0453-6

**Published:** 2020-06-17

**Authors:** Ke Yang, Yifan Chen, Kenneth Kin Wah To, Fang Wang, Delan Li, Likun Chen, Liwu Fu

**Affiliations:** 1 Collaborative Innovation Center for Cancer Medicine, State Key Laboratory of Oncology in South China, Sun Yat-sen University Cancer Center, Guangzhou, China; 2 Guangdong Esophageal Cancer Institute, Guangzhou, China; 3 School of Pharmacy, The Chinese University of Hong Kong, Hong Kong, China

**Keywords:** Cancer, Drug discovery

Correction to: *Experimental & Molecular Medicine*

10.1038/emm.2016.168 published online 17 March 2017

After online publication of this article, the authors noticed an error in Fig. 6e.

**Figure 1:**
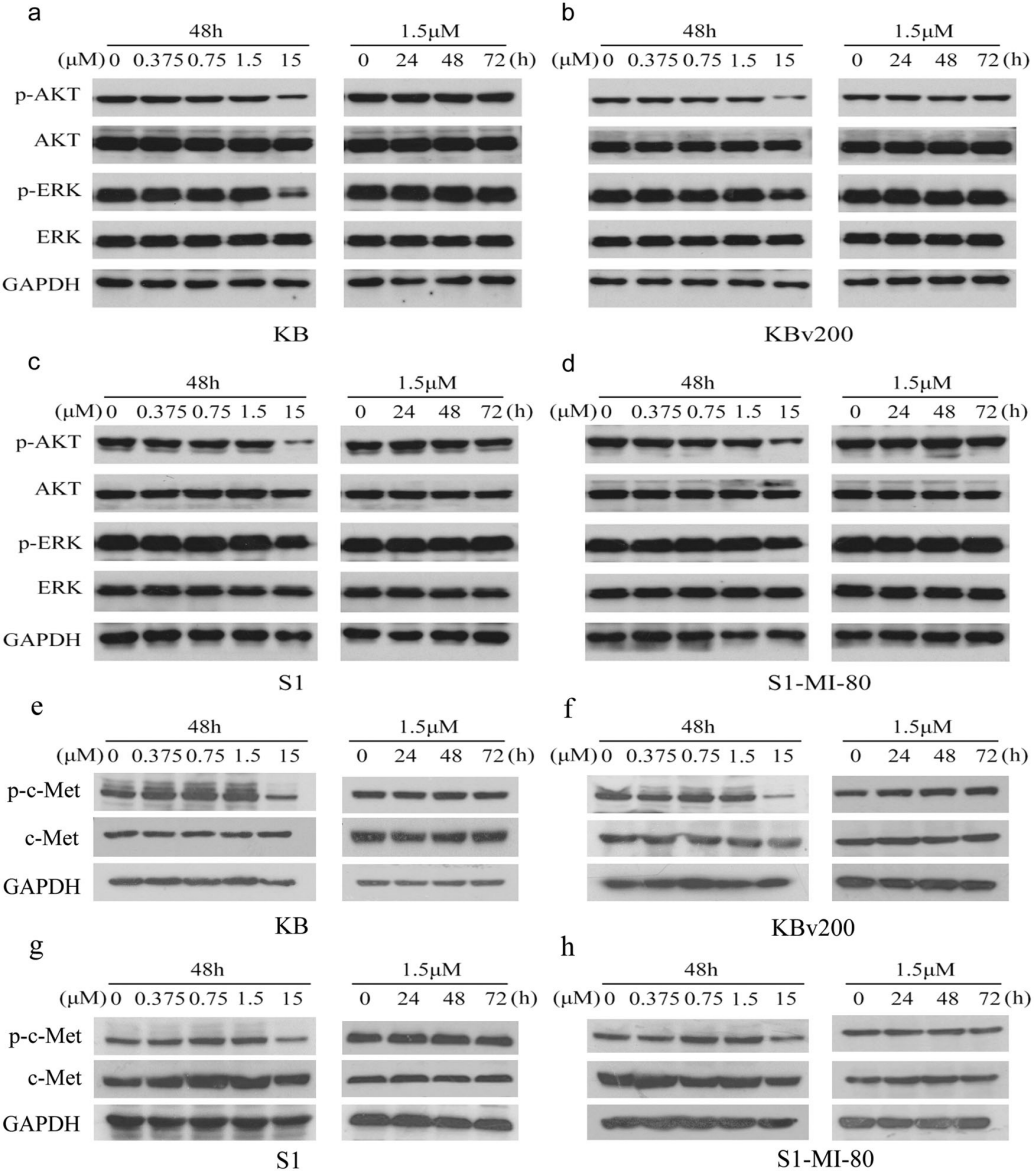
Fig. 6

In the version of this article originally published, Fig. 6e shows incorrect western blot bands of c-Met and GAPDH. The correct version is shown as Corrected Fig. 6e. The correction does not change the figure legends and the conclusion of the paper. The authors apologize for any inconvenience caused.

